# Insights into the Role of microRNAs in Colorectal Cancer (CRC) Metabolism

**DOI:** 10.3390/cancers12092462

**Published:** 2020-08-31

**Authors:** Kha Wai Hon, Syafiq Asnawi Zainal Abidin, Iekhsan Othman, Rakesh Naidu

**Affiliations:** Jeffrey Cheah School of Medicine and Health Sciences, Monash University Malaysia, Selangor Darul Ehsan 47500, Malaysia; kha.hon@monash.edu (K.W.H.); syafiq.asnawi@monash.edu (S.A.Z.A.); iekhsan.othman@monash.edu (I.O.)

**Keywords:** colorectal cancer, metabolism, miRNAs, metabolic reprogramming

## Abstract

**Simple Summary:**

Tumour cells have been shown to demonstrate changes in metabolic pathways as compared to normal cells. Colorectal cancer (CRC) is one of the most frequently diagnosed cancers globally. Some studies suggest that microRNAs (miRNAs) could play potential role in cancer cell metabolism. Our review aims to identify the role of different miRNAs in regulating the metabolism of CRC cells. Certain miRNAs could become potential biomarkers and even therapeutic targets based on their importance in CRC cell metabolism.

**Abstract:**

Colorectal cancer (CRC) is one of the most frequently diagnosed cancers, with a high mortality rate globally. The pathophysiology of CRC is mainly initiated by alteration in gene expression, leading to dysregulation in multiple signalling pathways and cellular processes. Metabolic reprogramming is one of the important cancer hallmarks in CRC, which involves the adaptive changes in tumour cell metabolism to sustain the high energy requirements for rapid cell proliferation. There are several mechanisms in the metabolic reprogramming of cancer cells, such as aerobic glycolysis, oxidative phosphorylation, lactate and fatty acids metabolism. MicroRNAs (miRNAs) are a class of non-coding RNAs that are responsible for post-transcriptional regulation of gene expression. Differential expression of miRNAs has been shown to play an important role in different aspects of tumorigenesis, such as proliferation, apoptosis, and drug resistance, as well as metabolic reprogramming. Increasing evidence also reports that miRNAs could function as potential regulators of metabolic reprogramming in CRC cells. This review provides an insight into the role of different miRNAs in regulating the metabolism of CRC cells as well as to discuss the potential role of miRNAs as biomarkers or therapeutic targets in CRC tumour metabolism.

## 1. Introduction

According to Global Cancer Statistics 2018 published by The International Agency for Research on Cancer (IARC), colorectal cancer (CRC) has emerged as the third most commonly diagnosed cancer in males and second in females [1]. CRC is also the second leading cause of cancer-related mortality in both sexes globally [1]. Additionally, the incidence and mortality rates of CRC are highly country-specific with wide geographical variation [2]. CRC originates from the epithelial cells lining the colon or rectum of the gastrointestinal tract, by forming benign polyps on the inner wall of the colon or rectum [3]. Later, some of the polyps become cancerous over time to develop into adenocarcinoma. As the malignancy progresses into the end stage, the tumour cells directly invade through the bowel wall into adjacent structures and metastasise to regional lymph nodes and distant organs through lymphatic and venous routes [4]. The pathophysiology of CRC is well known as being initiated by alteration in gene expression, which contributes towards dysregulation in signalling pathways and cellular processes, resulting in the development of tumour heterogeneity such as proliferation, metastasis and drug resistance [5,6,7].

**Figure 1:**
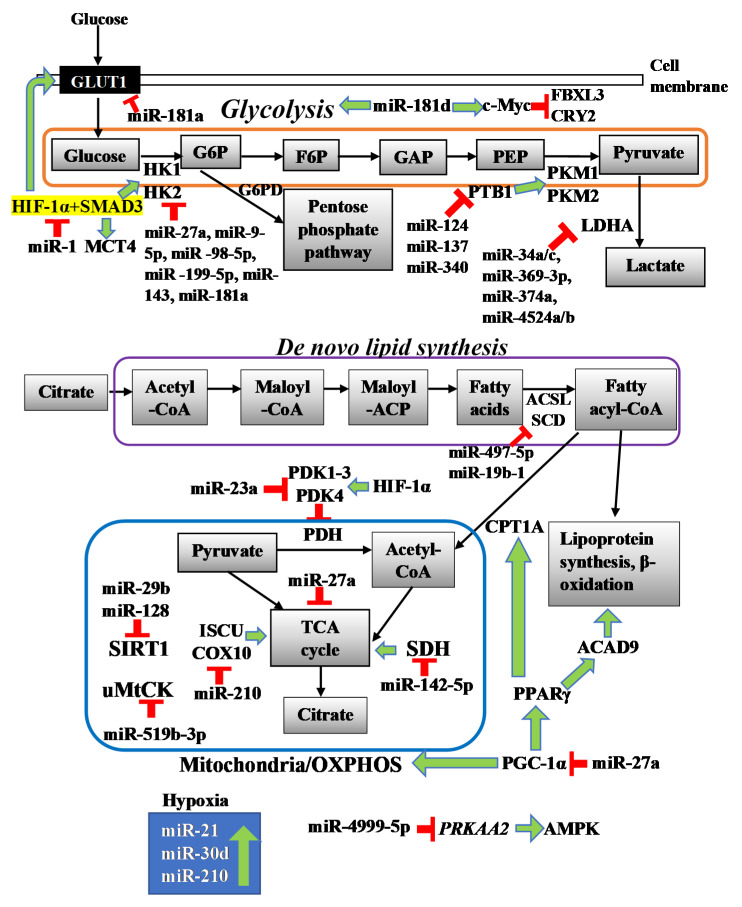
MicroRNAs modulate colorectal cancer (CRC) cell metabolism by targeting key metabolic enzymes and signalling pathways. Red sign indicates inhibition while green arrow indicates upregulation/promotion. G6P: glucose 6-phosphate, F6P: fructose 6-phosphate, GAP: glyceraldehyde 3-phosphate, PEP: phosphoenolpyruvate.

Metabolic reprogramming is an important cancer hallmark in CRC, which refers to the adaptive changes in tumour cell metabolism. Metabolic reprogramming can be regulated through several mechanisms including aerobic glycolysis, mitochondrial energy production, lactate, and fatty acid metabolism [8,9,10,11]. Thus, metabolic reprogramming fulfils the high energetic requirement of CRC cancer cells to sustain a rapid proliferation rate, even at low oxygen concentration with the use of alternative carbon sources. Increasing evidence has highlighted that microRNAs (miRNAs) are important regulators of metabolic reprogramming in CRC. MiRNAs are a class of small, endogenous non-coding RNAs (ncRNAs), with a single stranded structure of length between 19 and 23 nucleotides [12]. MiRNAs are known to carry out post-transcription gene regulation via complementary binding between seed region of miRNA and 3′-untranslated region (UTR) of target mRNA [13]. Depending on the degree of complementarity, miRNA can silence target mRNA through various mechanisms such as target cleavage, translational repression, and message degradation [14,15,16]. It has been estimated that the miRNAs are responsible for post-transcriptional regulation of nearly 60 percent of all human protein-coding genes, and therefore miRNAs were also described as the “master regulators of gene expression” in the previous literature [17,18]. Aberrant expression of miRNAs has been shown to play important roles in different aspects of tumorigenesis, such as proliferation, differentiation, apoptosis, and drug resistance, as well as metabolism [8,19,20,21]. Interaction between miRNAs and other biomolecules including enzymes, transporters, tumour suppressors and oncogenes is equally crucial to modulate metabolic reprogramming in cancer cells [22,23,24]. In this review, we focus on the different roles of miRNAs in regulating colorectal cancer metabolism. This review also provides insight into promising miRNAs as potential biomarkers or therapeutic targets in colorectal cancer. A summary of the miRNAs and key target metabolic enzymes as well as signalling pathways involved in the CRC cell metabolism are shown in Table 1 and Figure 1, respectively.

## 2. Role of miRNAs in Mitochondrial Metabolism/OXPHOS Metabolism

Glucose metabolism in human cells can be divided into two parts, which are glycolysis in cytosol and oxidative phosphorylation (OXPHOS) in mitochondria [42]. Once D-glucose is internalized into the cell, aerobic glycolysis is responsible for converting D-glucose into pyruvate which will enter OXPHOS in mitochondria later [42]. For most human cells, OXPHOS coupled with Krebs tricarboxylic acid (TCA) cycle in mitochondria are the main intracellular producers of energy in the form of adenosine triphosphate (ATP) [42]. When the amount of oxygen is limited, pyruvate can be converted into lactate in cytosol for alternative energy production [42]. Mitochondria in tumour cells is also responsible for the maintenance of cancer proliferation by converting available nutrients such as proteins and fatty acids into cellular components. Interestingly, tumour cells have been demonstrated to shift towards aerobic glycolysis and/or lactate metabolism upon metabolic reprogramming to sustain energy requirement, which will be discussed later in this review. However, tumour cells can alternate between OXPHOS metabolism and aerobic glycolysis to compensate for the high energetic requirement during growth and proliferation.

### 2.1. miR-23a

Deng et al. reported that overexpression of miR-23a in CRC cells indirectly promoted the activation of pyruvate dehydrogenase (PDH) involved in OXPHOS to generate sufficient ATP for tumour cell proliferation [25]. Upregulated miR-23a suppressed pyruvate dehydrogenase lipoamide kinase isozyme 4 (PDK4), which is a negative regulator of CRC proliferation via the inhibition of PDH [25]. Thus, miR-23a is essential for CRC cell proliferation by targeting PDK4 to release PDH for ATP production.

### 2.2. miR-519b-3p

Ubiquitous mitochondrial creatine kinase (uMtCK) is an isoenzyme in mitochondria, which catalyses the reversible transfer of phosphate groups from phosphocreatine to ADP, generating ATP and creatine [43]. Recently, uMtCK was identified as a direct target of miR-519b-3p in CRC [26]. Downregulation of miR-519b-3p in CRC tissue samples was negatively correlated with the level of uMtCK, which may promote the CRC development [26]. Further investigation also revealed that overexpression of miR-519b-3p negatively regulated uMtCK via Wnt signalling pathway in CRC cells [26]. Thus, miR-519b-3p was proposed as a novel target to inhibit CRC cell proliferation and invasion via uMtCK/Wnt signalling.

### 2.3. miR-142-5p

Succinate dehydrogenase (SDH) is an important respiratory enzyme located in the inner mitochondrial membrane that is involved in the TCA cycle and electron transport chain for oxidative phosphorylation [44]. There are four subunits of SDH complexes, which include SDHA, SDHB, SDHC, and SDHD [45]. Among all of these, downregulation of SDHB is associated with the regulation of Warburg effect in different cancers such as hepatocellular carcinoma [46] and CRC [47]. Liu et al. revealed that the up-regulation of miR-142-5p was correlated with the suppression of SDHB in CRC tissue [27]. In vitro analysis showed that miR-142-5p directly inhibited SDHB to promote aerobic glycolysis in CRC cells by reducing oxygen intake while increasing glucose consumption and lactate production [27]. Thus, the Warburg effect induced by miR-142-5p enhances the proliferation rate and colony formation ability of CRC cells [27].

### 2.4. miR-210

OXPHOS metabolism in mitochondria is also a major producer of reactive oxygen species (ROS) [42]. Regulation of the ROS level in cancer cells is crucial for the activation of oncogenic signalling pathways and metabolic reprogramming. Overexpression of miR-210 in CRC cells was reported to induce apoptosis by increasing the ROS production [8]. Previously, miR-210 was shown to suppress mitochondrial respiration in CRC cells under hypoxic condition, by targeting iron sulphur scaffold protein (ISCU) and COX 10, which are cofactors for key enzymes involved in TCA cycle [28,48]. MiR-210 also upregulated the ROS generation in CRC cells in response to hypoxia [28,48].

### 2.5. miR-29b and miR-128

Sirtuin 1 (SIRT1) is an enzyme of the NAD+-dependent histone deacetylase family, which is responsible for deacetylating stress-related transcription factors (such as PGC-1α in mitochondria) for the activation of antioxidant genes and the reduction in cellular ROS level [49]. Interestingly, SIRT1 was reported to be targeted by certain miRNAs to confer drug resistance in CRC cells by interfering with drug-induced ROS production and apoptosis. Liu et al. revealed that overexpression of miR-29b attenuated the oxaliplatin resistance in SW480 CRC cells by suppressing SIRT1 to promote ROS generation and apoptosis via activation of caspases 9, 7 and 3 [29]. Another study by Lian et al. also reported that miR-128 directly targeted SIRT1 to regulate the resistance of CRC cells towards tumour necrosis factor-related apoptosis-inducing ligand (TRAIL) which is an anti-cancer agent [30]. Their results demonstrate that overexpression of miR-128 in TRAIL-treated CRC cells suppressed SIRT1 expression, which promoted ROS production [30]. Subsequently, the increase in ROS level induced DR5 expression and increased TRAIL-induced apoptosis in CRC cells [30]. This shows that the ROS-miRNAs interaction could affect the drug resistance of CRC cells through the regulation of antioxidant enzymes and redox-sensitive signalling pathways.

### 2.6. miR-27a

More recently, Barisciano et al. presented a comprehensive work on the importance of miR-27a as a master regulator of metabolic reprogramming in CRC cells [31]. Previous work demonstrated that miR-27a is upregulated in CRC tissues and mostly associated with cell proliferation, tumour expansion and immunosuppression [50,51]. Apparently, miR-27a facilitates the mitochondrial activity and glycolysis as well as promoting drug resistance in CRC cells. The role of miR-27a in glycolysis of CRC cells will be discussed in another section. Knockdown of miR-27a in CRC cells increased the levels of citrate synthase activity (TCA cycle gatekeeper enzyme), dihydrolipoamide s-acetyltransferase (DLAT, the E2 component of PDH), intracellular ATP and ROS, which implicates the activation of mitochondrial respiration [31]. Knockdown of miR-27a also promoted the expression level of PPAR gamma co-activator-1α (PGC-1α) which regulates mitochondrial activity and biogenesis [31]. This shows that miR-27a controls mitochondrial metabolism directly via PGC-1α in CRC cells. In addition, PGC-1α also regulates PPARγ, which is responsible for transferring fatty acids into mitochondria for metabolism and degradation [52]. Downstream effectors of PPARγ, namely carnitine palmitoyl-transferase 1A (CPT1A) and Acyl-CoA dehydrogenase family member 9 (ACAD9), were predicted to be targeted by miR-27a. CPT1A is the acyl-CoA transporter into mitochondria during β-oxidation of unsaturated fatty acids, while ACAD9 is the rate-limiting enzyme in fatty acid β-oxidation [53,54]. The protein level of CPT1A and ACAD9 was upregulated in CRC cells upon suppression of miR-27a [31]. This suggests that miR-27a could indirectly regulate mitochondrial respiration via PPARγ.

## 3. Role of miRNAs in Glycolysis

As compared to normal cells, most of the cancer cells predominantly increase their glucose consumption with a rapid rate of aerobic glycolysis, to produce a high amount of intermediate glycolytic metabolites and pyruvate [55]. Metabolic reprogramming allows cancer cells to alter their ATP production from oxidative phosphorylation (TCA cycle) in mitochondria into a non-oxidative pathway led by aerobic glycolysis and enhanced lactate production [56]. Most of the pyruvate in cancer cells is converted into lactate for the use of the Krebs cycle in cytosol to produce ATP, instead of entering mitochondrial oxidative respiration [57]. This phenomenon is known as the Warburg effect [57,58]. The Warburg effect has been observed in a wide range of cancers, including CRC. Although the combined glucose metabolism of aerobic glycolysis and lactate secretion generates less ATP than OXPHOS, aerobic glycolysis can generate ATP more rapidly to fulfil the need of rapidly dividing cancer cells. This metabolic shift also provides cancer cells with the biosynthesis of more macromolecules such as nucleic acids, phospholipids and fatty acids that are essential for cell growth and maintenance [42,55]. In addition, less utilization of OXPHOS is also believed to protect cancer cells from the generation of ROS and oxidative stress during rapid cell proliferation.

Metabolic reprogramming of cancer cells into aerobic glycolysis involves the alteration of transmembrane glucose transporters (GLUTs) and glycolytic enzymes, such as hexokinase (HK), lactate dehydrogenase (LDH) and pyruvate kinase (PK), to accelerate glucose uptake and lactate production [42]. Hexokinase functions as an essential catalyst in the first irreversible step of glycolysis when glucose is phosphorylated to glucose-6-phosphate with the consumption of ATP [42]. There are four important isoforms of mammalian hexokinase, designated HK1–4; in particular, isoform 2 (HK2) is dysregulated in multiple cancers, including CRC [42,59,60].

### 3.1. miR-27a

As mentioned in a previous section, miR-27a facilitates mitochondrial respiration and glycolysis in metabolic reprogramming of CRC cells (39). Knockdown of miR-27a increased the expression level of HK1 and HK2 (39). This implies that miR-27a facilitates glycolysis by targeting HK1 and HK2.

### 3.2. miR-143

Additionally, miR-143 has been reported to be downregulated in CRC and negatively associated with cancer progression [61]. A previous study by Gregerson et al. identified hexokinase HK2 as direct target of miR-143 in colon cancer cells [32]. The authors revealed that overexpression of miR-143 suppressed the HK2 expression, and subsequently lactate production was significantly reduced in colon cancer cells [32]. Downregulation of miR-143 in colon cancer cells could be responsible for promoting the metabolic reprogramming towards aerobic glycolysis with the upregulation of HK2.

### 3.3. miR-9-5p, -98-5p, and -199-5p

The expression of HK2 could also be associated with other miRNAs, representing a complex regulatory network in the metabolic reprogramming of CRC cells. Snezhkina et al. performed comprehensive analysis on the data presented in The Cancer Genome Atlas (TCGA) and five miRNA–mRNA target interaction databases (TargetScan, DIANA microT, mirSVR (miRanda), PicTar and miRTarBase) to look for potential miRNAs that may inhibit HK2 expression in CRC [33]. Their validation using quantitative PCR on a set of CRC tissue samples showed that the overexpression of miR-9-5p, -98-5p, and -199-5p was correlated with downregulation of HK2 [33]. These three miRNAs could be involved in negative regulation of HK2 in CRC, although the underlying mechanism requires further investigation.

### 3.4. miR-181 Family

Wei et al. demonstrated that miR-181a can mediate the Warburg effect in CRC cells by targeting GLUT1 and HK2 via the PTEN/AKT pathway [34]. Their study showed that miR-181a was significantly overexpressed in CRC tissue [34]. Overexpression of miR-181a in CRC cell lines enhanced cell proliferation by upregulating the expression of GLUT1 and HK2 to increase glucose uptake and lactate production [34]. MiR-181a also inhibits PTEN to induce the phosphorylation of AKT, which is essential for the miR-181a-induced metabolic shift in CRC cells [34]. PTEN is a tumour suppressor commonly mutated in many cancers including glioblastoma [62], prostate [63], breast [64] and CRC [65]. AKT, also known as serine/threonine-specific protein kinase, is a proto-oncogene and downstream effector which has been associated with multiple signalling pathways and cellular metabolism in cancer cells [66,67,68]. Notably, PTEN is well studied as the negative upstream regulator of PI3K/AKT intracellular signalling axis in modulating tumorigenesis [69]. Suppression of PTEN activates the PI3K/AKT signalling pathway, which is an oncogenic pathway to promote cell proliferation/invasiveness and apoptosis [70,71]. Another member of miR-181 family, miR-181d, was reported to promote glycolysis in CRC cells via the c-Myc-miR-181d-CRY2/FBXL3 feed-forward loop [35]. MiR-181d was significantly overexpressed in CRC tissue and associated with glycolysis in CRC cells [35]. In addition, miR-181d also modulates the post-transcriptional regulation of c-Myc by suppressing FBXL3 and CRY2, which are responsible for ubiquitinate and degrade c-Myc cooperatively [72]. In cancers, c-Myc is an essential transcription factor that is frequently expressed to activate the expression of many genes involved in cell proliferation and metabolism [73]. This implies that c-Myc is required for miR-181d-induced glycolysis in CRC cells [74]. Activated c-Myc increases the expression of miR-181d and inhibits the transcription of FBXL3 and CRY2 in CRC cells, suggesting a feedback loop established to regulate glycolysis [35]. Thus, miR-181d acts as an oncomiR to promote aerobic glycolysis in CRC by protecting c-Myc from FBXL3 and CRY2-mediated degradation.

### 3.5. miR-1

MiR-1 is a well-known tumour suppressor that promotes tumour progression in multiple types of cancers, such as esophageal squamous cell carcinoma [75], CRC [76] and glioblastoma [77]. Xu et al. revealed that aberrant expression of miR-1 facilitated the aerobic glycolysis in CRC cells via the miR-1/SMAD3/HIF-1α axis to promote cancer progression [36]. The authors discovered that miR-1 suppressed CRC cell proliferation via the inhibition of glycolysis and negative regulation of SMAD3 activity and HIF-1α expression [36]. Their results show that miR-1 directly silenced HIF-1α upon binding, to downregulate glycolysis and inhibit proliferation [36]. HIF-1α (hypoxia inducible factor-1 alpha) is a key regulator of cancer cell proliferation that is activated in response to the hypoxic tumour microenvironment, to initiate the metabolic switch in cancer cells from oxidative phosphorylation to glycolysis [78]. HIF-1α promotes the expression of glucose transporters to increase glucose uptake while activating pyruvate dehydrogenase kinases (PDK1–3) to suppress pyruvate dehydrogenase and prevent pyruvate entering TCA cycle in mitochondria [78]. Xu et al. also revealed that miR-1 could terminate the interaction between SMAD3 and HIF-1α, leading to deactivation of HIF-1α and SMAD3 [36]. Subsequently, the expression of metabolic enzymes in the Warburg effect, such as HK2 and MCT4, is significantly reduced, affecting the rate of tumour proliferation [36]. SMAD3 is an important downstream component of TGF-β signalling, which can be activated upon phosphorylation to bind with promoter for gene translation of HIF-1α, HK2 and MCT4 [79].

### 3.6. miR-124, miR-137 and miR-340

Pyruvate kinase (PK) is the rate-limiting enzyme involved in the last step of glycolysis, catalysing the transphosphorylation between phosphoenolpyruvate (PEP) and adenosine diphosphate (ADP), to generate one molecule of pyruvate and one molecule of ATP [42]. In mammals, there are four isoforms of the PK family: liver-type PK (PKL), red blood cell PK (PKR), and PK muscle isozyme M1 and M2 (PKM1 and PKM2, respectively), each with tissue-specific expression [80]. PKM1 and PKM2 are generated by alternative splicing of primary RNA transcripts of the *PKM* gene, which spliced into sequences containing either exon 9 (PKM1) or exon 10 (PKM2), respectively [81]. PKM1 is expressed in most well-differentiated tissues and promotes oxidative phosphorylation, whereas PKM2 is exclusively expressed in rapid proliferating cells, such as embryonic cells and cancer cells, to promotes glycolysis even under aerobic conditions [82,83]. Interestingly, several miRNAs (miR-124, miR-137 and miR-340) were reported to counteract the Warburg effect in CRC cells through the switching of PKM isoform expression from PKM2 to PKM1 [11,37]. These miRNAs directly target polypyrimidine tract-binding protein 1 (PTB1), which is a splicer of the *PKM* gene that represses PKM1 and favours PKM2 synthesis [84,85]. Overexpression of miR-124, miR-137 and miR-340 inhibited PTB1 to stop the switching of PKM isoforms, leading to high ratios of PKM1/PKM2 in CRC cells [11,37]. Subsequently, the glycolysis rate was reduced significantly while oxidative phosphorylation was upregulated, resulting in apoptotic cell death and/or autophagy [11,37]. Therefore, miR-124, miR-137 and miR-340 function as tumour suppressors to modulate cell proliferation and Warburg effect in CRC through PTB1/PKM1/PKM2 cascade [11,37].

### 3.7. miR-4999-5p

5′ AMP-activated protein kinase (AMPK) is an essential enzyme that functions as an internal sensor of intracellular ATP levels and regulates cellular energy homeostasis mainly through the metabolism of glucose and fatty acids [86]. AMPK exists as a heterotrimeric protein complex that is made up of α, β, and γ subunits, while the α subunit can exist as either the α1 or α2 isoform [86]. *PRKAA2*, which is the gene that encodes AMPKα2, has been shown to modulate cell proliferation and signalling pathways in different cancers, including bladder [87], pancreas [88] and CRC [89]. Recently, miR-4999-5p has been reported to target *PRKAA2* to facilitate metabolic reprogramming in CRC cells [38]. MiR-4999-5p was highly expressed in CRC tissue samples and associated with poor survival outcome of CRC patients [38]. Overexpression of miR-4999-5p increased cell proliferation rate, glucose uptake, cellular G6P levels, and lactate production in CRC cells [38]. Correlation analysis and dual-luciferase reporter assays confirmed that miR-4999-5p was negatively correlated with *PRKAA2* in a direct manner [38]. Glycolysis rate and capacity were restored by *PRKAA2* knockdown in miR-4999-5p-silenced CRC cells [38]. These findings suggest that miR-4999-5p promotes CRC progression and glucose metabolic reprogramming via targeting *PRKAA2*.

## 4. Role of miRNAs in Lactate Metabolism

Upon metabolic reprogramming of tumour cells, pyruvate generated by glycolysis is mostly converted into lactate by the action of lactate dehydrogenase enzyme (LDH), instead of generating acetyl-CoA for OXPHOS metabolism in mitochondria [57]. Previous studies have shown that lactate dehydrogenase A (LDHA) is a key player of the Warburg effect in tumour cell metabolism, which catalyses the inter-conversion of pyruvate and L-lactate as well as NADH and NAD+ conversion, which are essential for the early steps of glycolysis [90,91,92,93,94].

### miR-34a, miR-34c, miR-369-3p, miR-374a, and miR-4524a/b

Wang et al. reported that LDHA was highly expressed in CRC tissue as compared to adjacent normal tissue, which may suggest the importance of LDHA in CRC pathogenesis [10]. Subsequently, the in vitro work also confirmed that LDHA was negatively regulated by a set of miRNAs (miR-34a, miR-34c, miR-369-3p, miR-374a, and miR-4524a/b) to suppress aerobic glycolysis and cell proliferation in CRC cells [10]. However, further investigation is essential to elucidate the underlying mechanism of these miRNAs in regulating LDHA.

## 5. Role of miRNAs in Lipid Metabolism

Dysregulation of lipid metabolism is another metabolic change commonly found in many different cancers, including CRC [95,96,97,98]. Lipid metabolism produces metabolites that are essential for membrane biogenesis and protein modifications. As compared to normal cells, most cancer cells alter lipid metabolism by upregulating fatty acids’ (FA) de novo synthesis and cholesterol synthesis pathways, to fulfil the cellular requirement in proliferation, progression, and metastasis [99]. Increasing evidence has revealed the regulatory role of miRNAs upon interaction with enzymes involved in lipid metabolism of cancer cells.

### 5.1. miR-497-5p

Acyl-CoA synthetase (ACSL) is responsible for catalysing the conversion of long chain fatty acids (FAs) to acyl-CoA, in which the five known ACSL isoforms in mammals, including ACSL1, ACSL3, ACSL4, ACSL5, and ACSL6, have been related to carcinogenesis [100]. For instance, CRC cells exhibited high expression levels of ACSL1, ACSL4 and ACSL5 in previous studies [101,102]. A recent work by Gharib et al. revealed that miR-497-5p targets ACSL5 to regulate lipid metabolism in CRC cells and mediate starvation-induced apoptosis [39]. Notably, miR-497-5p is a member of the miR-15/16/195/424/497 family that is frequently associated with cancers, while miR-497-5p mainly functions as a tumour suppressor [103,104,105,106]. Overexpression of miR-497-5p suppresses the level of ACSL5 in CRC cells both in vitro and in vivo, resulting in lower levels of lipoprotein and higher rate of apoptosis [39]. Hence, miR-497-5p could be targeted as a therapeutic strategy to modulate lipid metabolism in CRC.

### 5.2. miR-19b-1

Stearoyl-CoA desaturase (SCD) is the rate-limiting enzyme that catalyses the overall de novo synthesis of monounsaturated fatty acids from saturated FAs [107]. Previously, the ACSL/SCD axis has been identified as the pro-tumorigenic regulator of epithelial-mesenchymal transition (EMT) in CRC cells to promote migratory and invasive properties [95,108]. In another study, miR-544a, miR-142, and miR-19b-1 were identified as potential regulators of the ACSL/SCD axis in CRC tissue [40]. Further investigation revealed that miR-19b-1 could inhibit de novo lipogenesis in CRC cells by limiting the FA-activating enzymes (ACSLs and SCD), leading to a significant reduction in cell invasion [40]. MiR-19b-1 could become a potential non-invasive biomarker for CRC due to its strong association with better prognosis in CRC patients as well as the ability to inhibit CRC cell invasion through ACSL/SCD axis [40].

## 6. Hypoxia-Induced Metabolic Reprogramming

Hypoxia, which refers to low oxygen tension, is a common characteristic in the tumour microenvironment for most solid tumours. Cancer cells can adapt and survive within a hypoxic microenvironment by altering their gene expression profile and metabolic reprogramming. In CRC, hypoxia is often correlated with poor prognosis, advanced clinical features as well as resistance towards chemo- and radiotherapy [41,109].

### miR-21, miR-30d and miR-210

Nijhuis et al. highlighted the importance of hypoxia-induced miRNAs in metabolic reprogramming as well as resistance towards 5-fluorouracil (5-FU) in CRC cells [41]. MiR-210 was the most significantly upregulated across all six different CRC cell lines under normal and hypoxic conditions [41]. Similarly, miR-210 was significantly upregulated in hypoxic areas of CRC tissues and correlated positively with hypoxia marker CAIX, suggesting the potential of miR-210 as a hypoxic biomarker in CRC. Previous studies have addressed miR-210 as hypoxamiR-210, as it is consistently associated with hypoxia in many cancer types, including CRC [109,110]. Researchers also discovered that miR-21 and miR-30d were upregulated in both hypoxic and 5-FU resistant CRC cells [41]. Treatment with miR-21 and miR-30d antagonists sensitized hypoxic CRC cells to 5-FU [41]. All these findings support the assertion that miR-21 and miR-30d regulate metabolic reprogramming in hypoxic CRC cells towards drug resistance.

## 7. Crosstalk between miRNAs and ncRNAs in Metabolic Reprogramming of CRC

Interaction between miRNAs and other classes of ncRNAs such as long non-coding RNAs (lncRNAs) and circular RNAs (circRNAs) is equally important to modulating metabolic reprogramming in CRC cells. LncRNAs are a group of non-coding RNAs with lengths exceeding 200 nt, which often function as competing endogenous RNAs (ceRNAs) to regulate miRNA/mRNA axis and the production of downstream proteins [111]. LncRNAs are recognized as important regulators of cellular functions and tumour progression in different cancers, including CRC [112]. LncRNA HNF1A-AS1 has been identified as modulating cell migration, invasion, and glycolysis via miR-124/*MYO6* axis in CRC cells [113]. HNF1A-AS1 was significantly upregulated in CRC tissue and cell lines, whereas knockdown of HNF1A-AS1 also inhibited intracellular glycolysis in CRC cells [113]. Researchers also further identified miR-124 as a direct target of HNF1A-AS1, while the *MYO6* gene was directly inhibited by miR-124 [113]. High expression of *MYO6* (myosin VI) has been associated with tumour progression in multiple cancers, including prostate [114], CRC [115], breast [116] and stomach [117]. LncRNA HNF1A-AS1 indirectly promoted *MYO6* expression by sponging miR-124 to regulate aerobic glycolysis in CRC cells.

Meanwhile, circular RNAs (circRNAs) represent a subset of endogenous non-coding RNAs with a circular loop structure that is covalently closed from the 5′-end to the 3′-end [118,119]. As compared to linear RNAs, circRNAs are abundant, highly conserved in almost all eukaryotic cells, and more stable against enzymatic degradation [120,121]. CircRNAs are being widely studied as potential regulators of post-transcriptional gene expression, since certain circRNAs have been reported to inhibit miRNAs upon binding as well as interacting with RNA-binding proteins (RBPs) [118]. Interaction between circRNAs and miRNAs could be essential in metabolic reprogramming of CRC cells. Evidently, the circular RNA circDENND4C has been shown to facilitate glycolysis of CRC cells through miR-760/GLUT1 axis [122]. Both circDENND4C and GLUT1 were upregulated in CRC tissues and cell lines, while knockdown of circDENND4C or GLUT1 reduced the proliferation rate, migration rate, glucose uptake and lactate production of CRC cells [122]. MiR-760 was confirmed as a direct target of circDENND4C upon knockdown of circDENND4C in CRC cells. Upregulation of miR-760 also suppressed GLUT1 in CRC cells, resulting in lower glucose uptake and lactate production [122]. Thus, circDENND4C promotes the expression of GLUT1 by inhibiting miR-760 to increase the glycolysis rate of CRC cells.

In addition, exosomes derived from chemo-resistant CRC cells have been shown to transfer ciRS-122 into chemo-sensitive CRC cells, promoting glycolysis and drug resistance via miR-122/PKM2 axis [123]. Exosomes are a subclass of extracellular vesicles that can transfer multiple types of biological molecules, including nucleic acids, proteins, and lipids, between the cells, regardless of cell type [124,125]. The importance of exosomes as intercellular messengers could serve as an unchartered territory to discover potential biomarkers for CRC [126]. PKM2 was upregulated in oxaliplatin-resistant CRC cells as compared to oxaliplatin-sensitive CRC cells, also associated with enhanced glycolysis and ATP production in oxaliplatin-resistant cells [123]. PKM2 is dominant in CRC, and responsible for catalysing the final reaction in glycolysis for ATP production and pyruvate synthesis [82,84]. Among multiple circRNAs differently expressed between drug-resistant and sensitive CRC cells, ciRS-122 was predicted as a potential inhibitor of miR-122 [123]. Both in vitro and in vivo studies revealed that exosomes derived from oxaliplatin-resistant CRC cells transferred ciRS-122 into oxaliplatin-sensitive CRC cells, and subsequently the expression levels of ciRS-122 and PKM2 protein were upregulated in oxaliplatin-sensitive cells [123]. Simultaneously, miR-122 was significantly suppressed while glycolysis and drug resistance were further elevated in oxaliplatin-sensitive CRC cells upon uptake of oxaliplatin-resistant exosomes [123]. CiRS-122 promotes glycolysis in CRC cells by inhibiting miR-122 and upregulating PKM2 protein, which may contribute towards oxaliplatin-resistance.

## 8. The Role of miRNAs in the Crosstalk between Metabolism and Liver Metastasis in CRC

It has been estimated that about 50% of CRC patients eventually develop cancer metastasis at an advanced stage of the malignancy, in which the liver is the most common metastatic site [127]. Metastatic CRC (mCRC) is often associated with a poor clinical prognosis and low survival rate [2,127]. MiRNAs have widely been investigated as potential biomarkers or therapeutic targets in mCRC [128]. However, little is known about the role of miRNAs in the crosstalk between CRC metabolism and liver metastasis. MiRNA-181a could be an important regulator in CRC by modulating tumour metabolism and liver metastasis. As mentioned earlier in Section 3.4, miR-181a can mediate the Warburg effect in CRC cells by targeting GLUT1 and HK2 via the PTEN/AKT pathway [34]. On the other hand, Ji et al. investigated the miRNA expression profile of CRC tissue from patients with or without liver metastases [129]. Their study revealed that miR-181a was significantly upregulated in CRC tissue from patients with liver metastasis [129]. MiR-181a could serve as a prognostic biomarker for mCRC patients due to its localization in colorectal epithelial cells as well as strong correlation with distant metastasis and poor overall survival [129]. Overexpression of miR-181a in CRC cell lines also promoted the cell motility, invasion, epithelial-mesenchymal transition (EMT) and metastasis [129]. Further investigation also showed that miR-181a targeted the tumour suppressor *WIF-1* gene, which was negatively associated with CRC metastasis and poor prognosis [129]. Taken together, miR-181a plays crucial roles in CRC by regulating the Warburg effect in cancer metabolism and promoting liver metastasis.

## 9. Conclusions

CRC is the third most common malignancy worldwide, with high rates of morbidity and mortality. Continuous effort has been made to elucidate the molecular mechanisms underlying the progression of CRC for improving diagnosis and management of CRC patients. This review has highlighted the importance of miRNAs in the metabolic reprogramming of CRC. Metabolic reprogramming is one of the key hallmarks of cancer, while in CRC, this phenotype is mainly regulated through several major aspects, including the well-known Warburg effect, glycolysis, oxidative phosphorylation, TCA cycle, lactate metabolism and lipid metabolism. MiRNAs function as the important regulators of metabolic reprogramming in CRC, by promoting or inhibiting certain effectors in response to the environmental stress. MiRNA-mediated alteration in cancer metabolism also contributes towards other cancer phenotypes, such as proliferation and chemo-resistance. The interaction between miRNAs, other classes of ncRNAs and multiple signalling pathways is equally essential to facilitate the cancer metabolism in CRC. With the increasing knowledge on miRNAs and recent advancement in high throughput technologies, namely microarray and RNA-seq, miRNAs could serve as future biomarkers or even therapeutic options in CRC. The development of miRNA-targeted therapy such as miRNA mimics or inhibitor could be useful to regulate the activity of metabolic-related genes based on CRC progression. Nevertheless, there is still a wide knowledge gap in CRC metabolism and many questions are yet to be elucidated.

## Figures and Tables

**Table 1:** The list of miRNAs involved in metabolic reprogramming of colorectal cancer (CRC).

MiRNA	Up/Down-Regulation	Function	Target Gene/Pathway	Reference
miR-23a	Upregulation	Activate PDH in OXPHOS for ATP production	PDK4	[25]
miR-519b-3p	Downregulation	Promote OXPHOS metabolism and cell proliferation	uMtCK/Wnt signalling	[26]
miR-142-5p	Upregulation	Promote aerobic glycolysis and Warburg effect	SDH	[27]
miR-210	Upregulation	Increase ROS production and suppress mitochondrial respiration	ISCU, COX 10	[8,28]
miR-29b	Upregulation	Promote ROS generation and apoptosis	SIRT1, Caspase 9, 7 and 3	[29]
miR-128	Upregulation	Promote ROS generation and apoptosis	SIRT1	[30]
miR-27a	Upregulation	Suppress mitochondrial respiration	PGC-1α, PPARγ, CPT1A and ACAD9	[31]
		Facilitate glycolysis	HK1, HK2	
miR-143	Downregulation	Promote aerobic glycolysis	HK2	[32]
miR-9-5p, -98-5p, and -199-5p	Upregulation	Facilitate aerobic glycolysis	HK2	[33]
miR-181a	Upregulation	Increase glucose uptake and lactate production	GLUT1 and HK2 via PTEN/AKT pathway	[34]
miR-181d	Upregulation	Promote aerobic glycolysis	c-Myc, CRY2, FBXL3	[35]
miR-1	Downregulation	Promote aerobic glycolysis	HIF-1α and SMAD3	[36]
miR-124, miR-137 and miR-340	Upregulation	Inhibit aerobic glycolysis	PTB1/PKM1/PKM2 cascade	[11,37]
miR-4999-5p	Upregulation	Increase glucose uptake and lactate production	*PRKAA2*	[38]
miR-34a, miR-34c, miR-369-3p, miR-374a, and miR-4524a/b	Upregulation	Suppress glycolysis and lactate production	LDHA	[10]
miR-497-5p	Downregulation	Promote lipid metabolism	ACSL5	[39]
miR-19b-1	Upregulation	Inhibit de novo lipogenesis	ACSL/SCD	[40]
miR-21, miR-30d and miR-210	Upregulation	Potential biomarker for hypoxia	-	[41]
